# Periodicity of molecular clusters based on symmetry-adapted orbital model

**DOI:** 10.1038/s41467-019-11649-0

**Published:** 2019-08-19

**Authors:** Takamasa Tsukamoto, Naoki Haruta, Tetsuya Kambe, Akiyoshi Kuzume, Kimihisa Yamamoto

**Affiliations:** 1 Institute of Innovative Research, Tokyo Institute of Technology, Yokohama, 226-8503 Japan; 2 ERATO, JST, Kawaguchi, Saitama, 332-0012 Japan; 3 Fukui Institute for Fundamental Chemistry, Kyoto University, Sakyou-ku Kyoto, 606-8103 Japan

**Keywords:** Nanoparticles, Computational chemistry

## Abstract

The periodic table has always contributed to the discovery of a number of elements. Is there no such principle for larger-scale substances than atoms? Many stable substances such as clusters have been predicted based on the jellium model, which usually assumes that their structures are approximately spherical. The jellium model is effective to explain subglobular clusters such as icosahedral clusters. To broaden the scope of this model, we propose the symmetry-adapted orbital model, which explicitly takes into account the level splittings of the electronic orbitals due to lower structural symmetries. This refinement indicates the possibility of an abundance of stable clusters with various shapes that obey a certain periodicity. Many existing substances are also governed by the same rule. Consequently, all substances with the same symmetry can be unified into a periodic framework in analogy to the periodic table of elements, which will act as a useful compass to find missing substances.

## Abstract (web summary)

Prediction of stable clusters based on the jellium model is limited because of the usual assumption of their spherical symmetry. Here the authors propose a symmetry adapted orbital model by quantum chemistry calculations for the prediction of stable clusters with various shapes that obey a certain periodicity.

## Introduction

In 1869, Mendeleev proposed the periodic table of the elements, which has long been the foundation of the natural sciences^1^. The periodic table has contributed to the discovery of a number of the elements that obey a certain periodic rule. Thereafter, much attention has been paid to higher-order substances that consist of the elements, including molecules, clusters, nanoparticles, and bulk substances. Although such substances have tremendous potential to be functional materials, it remains a difficult problem to predict and design unknown substances among the infinite combinations of constitutive elements. As with Mendeleev’s periodic table, such a periodic table for higher-order substances would be considerably valuable for a wide range of materials science. Is there no principle to govern larger-scale substances?

Recently, Tomalia and Khanna^2^ pointed out the possibility of nanoperiodicity in higher-order substances typified by superatoms that function as building blocks of higher-order architectures^3–5^ as with atoms that form molecules. It is known that some particularly stable clusters exhibit properties that resemble specific chemical elements in the periodic table, e.g., [Al_13_]^−^ and [Al_7_C]^−^^6,7^. Such clusters are referred to as superatoms, which have electronic structures similar to those of atoms^2,6–12^. The superatoms have been predicted on the basis of the jellium model^1,13^, which usually assumes that these structures have an approximately spherical symmetry. As described by the jellium model, molecular orbitals generated by valence electrons in highly symmetric clusters have shapes just like atomic orbitals. Accordingly, a cluster will be stable when the number of valence electrons coincides with a closed-shell structure based on the jellium model. This spherical approximation is applicable especially for subglobular *I*_*h*_ symmetric clusters. The initially discovered superatom was the [Al_13_]^−^ cluster with *I*_*h*_ symmetry^6^.

A theoretical framework that describes a broad range of substances is necessary to obtain a universal law. The spherical jellium model is a candidate for such a theory; however, most clusters do not have quasi-spherical *I*_*h*_ symmetry^14,15^, because their atomicities are inappropriate for the construction of *I*_*h*_ symmetry structures. Even if they have the icosahedral atomicities, many of the clusters exhibit Jahn–Teller or pseudo Jahn–Teller instability^16^ due to electronic reasons. On the other hand, particularly stable clusters without *I*_*h*_ symmetry have also been found, such as Au_20_ with *T*_*d*_ symmetry^8^. In such clusters, the validity of the spherical jellium model decreases relatively due to the split and shift of the superatomic orbital levels. Therefore, it is strongly desired to establish a more sophisticated method to find an abundance of stable clusters. We have focused on the violation of the spherical jellium model to extend the theory for the prediction of clusters. In the present study, we demonstrate that various stable clusters can be correctly predicted by considering their structural symmetry, in addition to the number of valence electrons and the number of constitutive atoms. This is the essence of our theory, the symmetry-adapted orbital model (SAO model) to reveal the hidden periodicity behind larger-scale substances.

## Results

### Symmetry-adapted orbital model

The SAO model is introduced as follows. Detailed formulation is given in the Supplementary Notes 1 and 2. Herein we discuss three types of structural symmetries based on Platonic solids: *I*_*h*_, *O*_*h*_, and *T*_*d*_. Superatomic orbitals derived from the spherical jellium model typically split under these structural symmetries. As shown in Table 1, the splitting pattern is completely determined within the point-group theory^17,18^. In the case of *I*_*h*_ symmetry, the *F* and *G* orbitals split into *T*_2*u*_ and *G*_*u*_ orbitals, as well as *G*_*g*_ and *H*_*g*_ orbitals, respectively. For the *O*_*h*_ symmetry, the *D* orbitals split into *E*_*g*_ and *T*_2*g*_ orbitals. The *F* and *G* orbitals split in a more complicated way. For the *T*_*d*_ symmetry, the splitting pattern is almost the same as that for *O*_*h*_.

**Table 1:** Splitting patterns of superatomic orbitals by symmetry lowering^17^

Spherical	*I*_*h*_	*O*_*h*_	*T*_*d*_
*S*	*A*_*g*_	*A*_1 *g*_	*A*_1_
*P*	*T*_1 *u*_	*T*_1 *u*_	*T*_2_
*D*	*H*_*g*_	*E*_*g*_⊕ *T*_2*g*_	*E* ⊕ *T*_2_
*F*	*T*_2*u*_ ⊕ *G*_*u*_	*A*_2*u*_ ⊕ *T*_1*u*_ ⊕ *T*_2*u*_	*A*_1_ ⊕ *T*_1_ ⊕ *T*_2_
*G*	*G*_*g*_ ⊕ *H*_*g*_	*A*_1*g*_ ⊕ *E*_*g*_ ⊕ *T*_1*g*_ ⊕ *T*_2*g*_	*A*_1_ ⊕ *E* ⊕ *T*_1_ ⊕ *T*_2_
*H*	*T*_1*u*_ ⊕ *T*_2*u*_ ⊕ *H*_*u*_	*E*_*u*_ ⊕ 2*T*_1*u*_ ⊕ *T*_2*u*_	*E* ⊕ *T*_1_ ⊕ 2*T*_2_

**Fig. 1:**
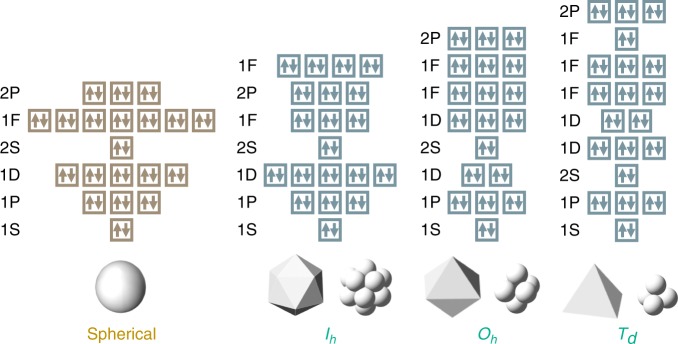
Electronic configurations based on the symmetry-adapted orbital (SAO) models. The order of the orbital levels generally obeys this rule, although many valence electron systems have strong interelectronic interactions and distorted structures, often changing the order of orbital levels partially

However, the order of orbital levels is not determined by the point-group theory^17,18^, but is dependent on the degree of level splittings. According to our quantum chemical calculations, the order of the orbital levels generally obeys a certain law with respect to each structural symmetry (Fig. 1). The reason is as follows. Let us take the spherical jellium model as the unperturbed system, as described in the Supplementary Notes 1 and 2. The perturbation of the nuclear charge distribution gives rise to the shift and split of the jellium orbital levels. According to the first-order perturbation theory, the jellium orbitals that overlap the nuclear charge distribution are selectively stabilized, depending on each structural symmetry, as compared with the spherical jellium model^19^. When the split orbitals are filled with a suitable number of valence electrons, structural symmetry is maintained without any Jahn–Teller distortion^16^. According to this model, the magic numbers, or the number of valence electrons required for full occupation of the split orbitals, are dependent on the structural symmetries. The magic numbers for each symmetry are listed in Table 2 (see also Fig. 1). It should also be noted that atomicity must be appropriate for the construction of each symmetric structure. The present SAO model is quite simple but more effective than expected, as revealed later with diverse examples.

**Table 2:** Magic numbers of valence electrons based on the SAO models

Magic number of valence electrons									
Spherical	2 (1*S*)^2^	8 (1*P*)^6^		18 (1*D*)^10^	20 (2*S*)^2^			34 (1*F*)^14^	40 (2*P*)^6^
*I*_*h*_	2 (1*S*)^2^	8 (1*P*)^6^		18 (1*D*)^10^	20 (2*S*)^2^	26 (1*F*^a^)^6^	32 (2*P*)^6^		40 (1*F*^a^)^8^
*O*_*h*_	2 (1*S*)^2^	8 (1*P*)^6^	12 (1*D*^a^)^4^	14 (2*S*)^2^	20 (1*D*^a^)^6^	26 (1*F*^a^)^6^	32 (2*P*)^6^	38 (1*F*^a^)^6^	
*T*_*d*_	2 (1*S*)^2^	8 (1*P*)^6^	10 (2*S*)^2^	16 (1*D*^a^)^6^	20 (1*D*^a^)^4^	26 (1*F*^a^)^6^	32 (1*F*^a^)^6^	34 (1*F*^a^)^2^	40 (2*P*)^6^

^a^Denotes the split orbitals. It should also be noted that atomicity must be appropriate for the construction of each symmetric structure

### First-principles calculations

**Fig. 2:**
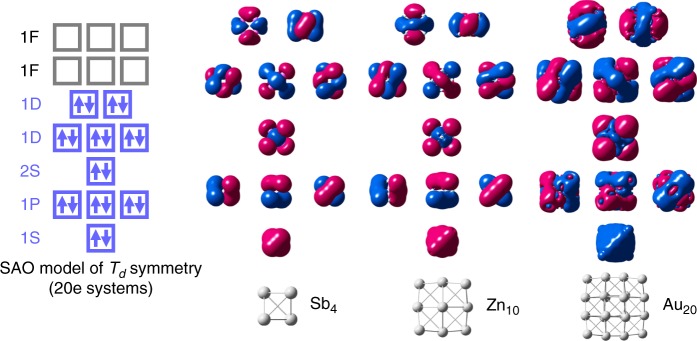
Molecular orbitals of *T*_*d*_-type clusters. They can be ascribed to the split superatomic orbitals, depending on the orbital angular momenta

**Fig. 3:**
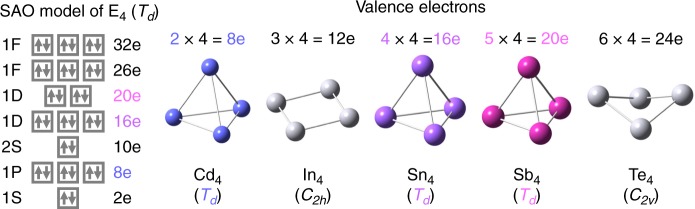
Optimized structures of *E*_4_ clusters with various valence electrons. The symmetry-adapted orbital (SAO)-based electronic configuration is also shown for reference

The SAO model was verified by comparison with the density functional theory (DFT) calculations, using the *T*_*d*_-type clusters as an example. Figure 2 shows that the molecular orbitals of the *T*_*d*_-type clusters can be ascribed to the split superatomic orbitals. According to the SAO model, in the case of four-atom clusters (*E*_4_), only the elements having 2, 4, and 5 valence electrons fill the split orbitals. For 10-atom and 20-atom clusters (*E*_10_ and *E*_20_), elements with one valence electron are also allowed. DFT calculations were performed for *E*_4_ clusters comprised of typical elements in the same period, as shown in Fig. 3. The elements Cd, In, Sn, Sb, and Te have 2, 3, 4, 5, and 6 valence electrons, respectively. It was confirmed that the *E*_4_ clusters are stable with *T*_*d*_ symmetry only for the elements with 2, 4, and 5 valence electrons. These elements coincide with full occupations of the SAOs which correspond to 1*P* (*T*_2_), 1*D* (*T*_2_), and 1*D* (*E*), respectively. No imaginary frequencies were observed in any of these clusters. On the other hand, the In_4_ and Te_4_ clusters are distorted due to the Jahn–Teller effect^16^, which originates from the partial fillings of the degenerate SAOs. However, suitable charge control allows these clusters to conserve their *T*_*d*_ symmetry, such as [In_4_]^2+^ and [Te_4_]^4+^ (Supplementary Figs. 1 and 2). These results clearly illustrate that the prediction of stable clusters by the SAO model is well consistent with the DFT calculations.

**Fig. 4:**
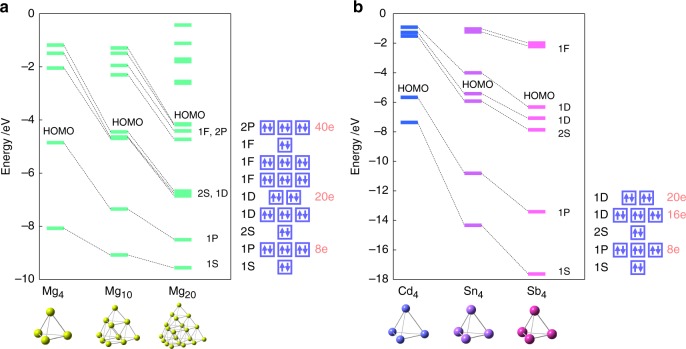
Orbital levels of different-sized and anisoelectronic clusters. **a** Mg_4_, Mg_10_, and Mg_20_. **b** Cd_4_, Sn_4_, and Sb_4_. The symmetry-adapted orbital (SAO)-based electronic configurations are also shown for reference

In the SAO model, there are some parameters that can control the electronic properties while conserving the structural symmetry. One parameter is the number of constitutive atoms: *E*_4_, *E*_10_, *E*_20_, *E*_35_, etc. The optimized geometries and molecular orbitals of Mg_4_, Mg_10_, and Mg_20_ are shown in Fig. 4a (see also Supplementary Figs. 3 and 4 for reference). Their 8, 20, and 40 total valence electrons fill the 1*P* (*T*_2_), 1*D* (*E*), and 2*P* (*T*_2_) orbitals, respectively. According to Fig. 4a, when the number of constitutive atoms increases, the jellium orbitals overlap more nuclear charges, and thereby become more stabilized. The sizes of the superatomic orbitals can also be controlled by this method. As the atomicity increases, the gap between the highest occupied molecular orbital (HOMO) and the lowest unoccupied molecular orbital (LUMO) becomes smaller and approaches that of the bulk state.

**Fig. 5:**
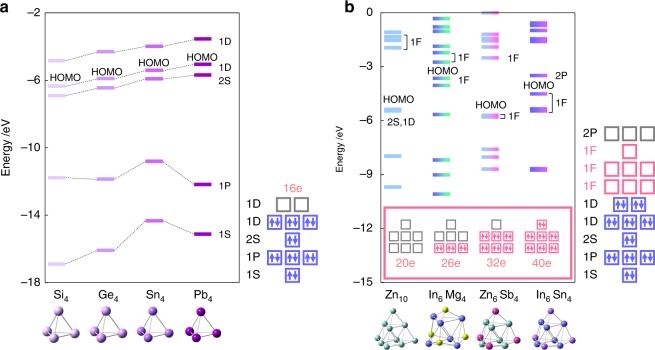
Orbital levels of isoelectronic and alloy clusters. **a** Si_4_, Ge_4_, Sn_4_, and Pb_4_. **b** Zn_10_, In_6_Mg_4_, Zn_6_Sb_4_, and In_6_Sn_4_. The symmetry-adapted orbital (SAO)-based electronic configurations are also shown for reference

Another parameter is the constitutive elements. The optimized geometries and molecular orbitals of Cd_4_, Sn_4_, and Sb_4_, which constitute elements in the same period, are shown in Fig. 4b (see also Supplementary Fig. 5 for reference). When the constitutive elements are different between two clusters, the orbital levels of the clusters are also different, because the effective potentials acting on valence electrons are different. The optimized geometries and molecular orbitals of Si_4_, Ge_4_, Sn_4_, and Pb_4_, which are Group 14 elements, are shown in Fig. 5a (see also Supplementary Figs. 6–8 for reference). These elements have 16 valence electrons, which coincide with the same orbital-filling condition. The orbital levels of heavier atoms tend to be less stabilized. The SAO model is also applicable to alloy clusters. Various compositions of elements enable the number of valence electrons to be precisely controlled. Figure 5b shows that the number of electrons that occupy the 1*F* superatomic orbitals can be controlled using alloy clusters. This example is regarded as mimicry of the lanthanide elements. The Pd atom, of which the electronic configuration is [Kr]4*d*^10^, can be utilized as a dummy element with 0 valence electrons. This technique is also applicable for control of the number of valence electrons (Supplementary Figs. 9 and 10). All of these examples possess *T*_*d*_ symmetry without the Jahn–Teller or pseudo Jahn–Teller instability^16^. These computational results thus validate the SAO model.

## Discussion

**Fig. 6:**
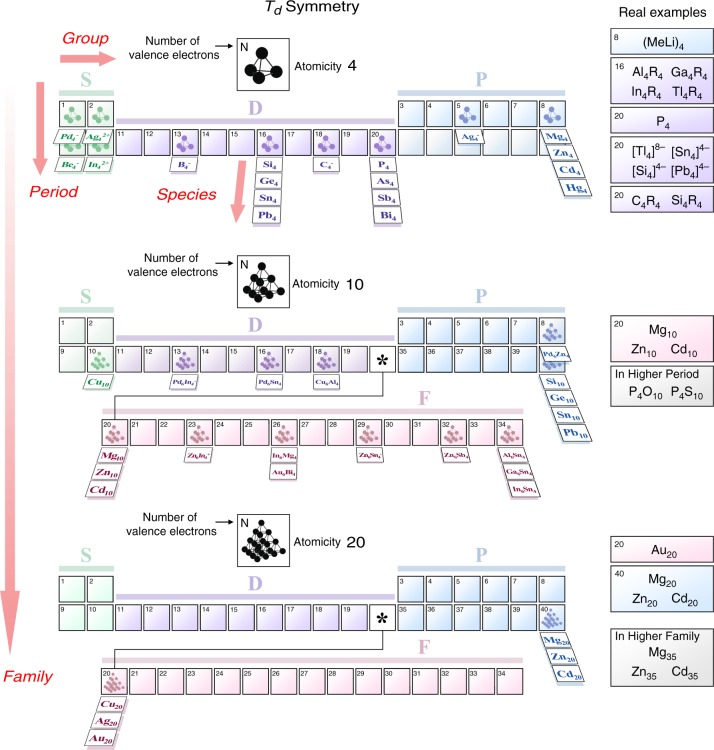
The periodic framework based on the *T*_*d*_-type symmetry-adapted orbital (SAO) model. Groups and periods are defined according to the outer-shell configurations. Families and species classify clusters from the viewpoints of the number of constitutive atoms and the constitutive elements, respectively

The calculated clusters can be classified in a periodic framework, in analogy to the periodic table of the elements (Fig. 6). Each piece in the framework can be regarded as an “element”. This framework not only has “groups” and “periods” but also “families” and “species” as new dimensions. In a similar way to the conventional periodic table, the groups and periods are defined according to the outer-shell configurations (see Cd_4_, Sn_4,_ and Sb_4_ as examples). Thus, there are *S*-, *P-*, *D-*, and *F*-blocks. Further, families classify clusters with the same structural symmetry from the viewpoint of the number of constitutive atoms. For example, Cu_10_, Mg_10_, and Ge_10_ belong to the same family X_10_, whereas Mg_4_, Mg_10_, and Mg_20_ belong to different families X_4_, X_10_, and X_20_, respectively. Species classify clusters with the same structure and the same total number of valence electrons from the viewpoint of the constitutive elements. For example, Si_10_, Ge_10_, Sn_10_, and Pb_10_ are all tetrahedral with 40 valence electrons but belong to different species. In this way, the SAO model directs a spotlight on the multi-dimensional periodicity behind an abundance of clusters.

**Fig. 7:**
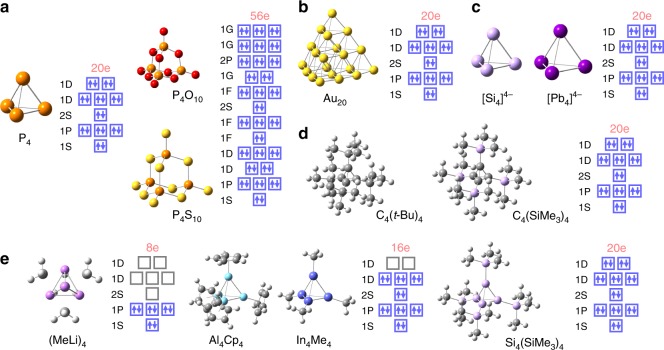
Electronic configurations of real compounds. **a** Natural products (P_4_, P_4_O_10_, and P_4_S_10_). **b** The magic gold cluster (Au_20_). **c** Zintl anions ([Si_4_]^4−^ and [Pb_4_]^4−^). **d** Organic compounds (C_4_(*t*-Bu)_4_ and C_4_(SiMe_3_)_4_). **e** Organometallic clusters ((MeLi)_4_, Al_4_Cp_4_, In_4_Me_4_, and Si_4_(SiMe_3_)_4_)

Many real compounds with *T*_*d*_ symmetry also support the SAO model. Figure 7a shows natural white phosphorus (P_4_)^20^ with its pentoxide and pentasulfide (P_4_O_10_ and P_4_S_10_)^21^. Figure 7b shows the famous gold cluster (Au_20_)^8^. All of these substances fulfill the orbital-filling conditions of the SAO model. As for P_4_O_10_ and P_4_S_10_, their central frameworks (P_4_O_6_ and P_4_S_6_) were used to count the number of valence electrons (see Supplementary Fig. 11). In the gas phase, the Mg_*N*_, Zn_*N*_, and Cd_*N*_ (*N* = 10, 20, and 35) clusters have been detected using mass spectrometry^22–25^. These atomicities are suitable only for the construction of *T*_*d*_ symmetric structures and the number of valence electrons coincides with the orbital-filling conditions of the SAO model (Fig. 4 and Supplementary Fig. 4). Figure 7c shows Zintl anions^26–28^ that fit the SAO model, e.g., [Si_4_]^4−^, [Sn_4_]^4−^, [Pb_4_]^4−^, and [Tl_4_]^8−^ (see also Supplementary Fig. 12). Some organic compounds such as tetrahedranes^29,30^ also satisfy the SAO model (Fig. 7d and Supplementary Fig. 13) due to one-electron donation from each substituent. Organometallic clusters also follow the SAO model. The alkyllithium tetramer (RLi)_4_^31^ is a good example of the SAO model (Fig. 7e and Supplementary Fig. 14); it has a total of eight electrons consistent with the SAO model. The stabilities of M_4_R_4_ (M = Al, Ga, In, and Tl)^32,33^ can be justified with the SAO model (Fig. 7e and Supplementary Fig. 15). Finally, *tetrahedro*-tetrasilane is also governed by the SAO model^34^ (Fig. 7e).

The SAO model can be applied to the *O*_*h*_ and *I*_*h*_ symmetries in the same way as that for the *T*_*d*_ symmetry. This means that various similar frameworks exist, depending on the symmetry of the clusters (Supplementary Figs. 16 and 17). We herein should note that highly symmetric clusters typified by *I*_*h*_ have fewer examples than tetrahedral clusters. This is because it is hard to satisfy the magic-number requirement of valence electrons, as well as that of constitutive atoms simultaneously. For example, it can be easily imagined that the number of valence electrons in a 13-atom monoelemental icosahedral cluster is limited to be multiples of 13. On the other hand, it should also be noted that there is more than one structural framework belonging to each point group. For instance, both the octahedron and cuboctahedron have the same *O*_*h*_ symmetry. The investigation of frameworks other than the present cases will thus lead to the discovery of novel periodic classifications of clusters.

Various innovative materials could be synthesized based on the SAO model (Supplementary Figs. 18 and 19). If the number of occupying electrons matches the degree of the degeneracy of the high-lying SAOs, then a spin arrangement is induced by exchange interactions, without Jahn–Teller instability^16^. In fact, [Na_55_]^+^ has been experimentally reported to possess subshell high spins^35^. Magnetic clusters with only non-magnetic elements could be designed and classified in the D-block, e.g., Cu_6_Al_4_ (Supplementary Fig. 20). We surmise that the lightest magnetic compounds without heavy *d* elements will be predicted by this approach. On the other hand, clusters classified in the F-blocks could behave as a photofunctional materials similar to the lanthanides (Fig. 5b and Supplementary Fig. 21).

A variety of clusters predicted by the SAO model are expected to be actually synthesized. Laser vaporization techniques combined with time-of-flight mass spectrometry produce many of them in the gas phase. To use the produced clusters as materials, they could be soft-landed onto self-assembled monolayers^36^. In the liquid phase, template-based synthetic methods are useful for the fabrication of clusters^37^. The isolation and crystallization of clusters could be realized by ligand protection^38^. Crystallization techniques have been established for the preparation of Zintl-type clusters^39^. The cocrystallization method for clusters and fullerenes would also be effective to obtain solid-state materials^5^.

In summary, we have proposed a symmetry-adapted orbital model that is beneficial for the prediction and design of stable clusters. This model indicates that an abundance of stable clusters could be classified as elements in a periodic framework, with respect to each structural symmetry. The periodic framework is strongly supported by many quantum chemical calculations and various existing substances. It is not only conceptual but also practical for the systematic exploration of unknown stable clusters. Among the infinite combinations of constitutive elements, this approach will be a significant contribution to the formation of innovative materials based on clusters with magnetic, optical, and catalytic functions.

## Methods

### First-principles calculations

Geometry optimizations and vibrational analyses were conducted using DFT calculations implemented in the Gaussian 09 package, Revision E.01^40^. The B3LYP functional and LanL2DZ basis set were employed. The 6-31G(d,p) basis set was used only for H, Li, and C. Typical elements with *s* and *p* valence electrons in their outermost shell are known to be suitable for the formation of superatomic orbitals. The *T*_*d*_ symmetric clusters comprised of typical elements were calculated with respect to the various atomicities (*E*_4_, *E*_10_, and *E*_20_). The *s*- and *p*-type valence electrons of the elements were counted as 1 for Li, Cu, Ag, and Au, 2 for Be, Mg, Zn, Cd, and Hg, 3 for B, Al, Ga, In, and Tl, 4 for C, Si, Ge, Sn, and Pb, 5 for P, As, Sb, and Bi, 6 for O, S, Se, and Te, 7 for I, and 0 for Pd. It was confirmed that all the obtained structures have no imaginary frequencies. A small part of the present clusters has also been discussed in our recent paper^41^, including Mg_4_, Mg_10_, Mg_20_, Zn_4_, Zn_10_, Zn_20_, Cd_4_, Cd_10_, Cd_20_, Si_10_, Ge_10_, Sn_10_, Pb_10_, Al_6_Sn_4_, Ga_6_Sn_4_, and In_6_Sn_4_.

## Supplementary information

Supplementary Information

## Data Availability

The data that support the findings of this study are available from the corresponding author upon reasonable request.

## Code availability

The Gaussian 09 package, Revision E.01^40^ was employed for all the electronic state calculations. The software and instructions for its use are available at http://gaussian.com/.
