# Supplementary material for: Periodicity of molecular clusters based on symmetry-adapted orbital model
Source: Nat Commun. 2019 Aug 19;10:3727. doi: 10.1038/s41467-019-11649-0 (PMC6700083; doi:10.1038/s41467-019-11649-0)
Supplement: Supplementary file 1 — Supplementary Information [file 41467_2019_11649_MOESM1_ESM.pdf]

# Supplementary Information

# Periodicity of molecular clusters

# based on symmetry-adapted orbital model

Tsukamoto *et al.*

## Supplementary Note 1 Spherical Jellium Model

Before describing the present symmetry-adapted orbital model, we briefly review the conventional jellium model to make this document self-contained. In the spherical jellium model, the motion of an electron in the infinite spherical potential well, $v(r)$, is discussed,

$$v(r) = \begin{cases} 0 & (r \leq a), \\ \infty & (r > a), \end{cases} \tag{1}$$

where $a$ is a radius of the spherical potential well, which is regarded as the size of a metal cluster in practical. The spherical coordinate system is employed here for the description of the electron,

$$(x,y,z) = (r\sin\theta\cos\varphi, r\sin\theta\sin\varphi, r\cos\theta), \quad 0 \leq r < \infty, \quad 0 \leq \theta \leq \pi, \quad 0 \leq \varphi < 2\pi. \tag{2}$$

Its Hamiltonian $\hat{h}$ is given by

$$\hat{h} = -\frac{\hbar^2}{2m_e}\nabla^2 + v(r) = -\frac{\hbar^2}{2m_e}\left[\frac{\partial^2}{\partial r^2} + \frac{2}{r}\frac{\partial}{\partial r} - \frac{\hat{\boldsymbol{l}}^2}{\hbar^2 r^2}\right] + v(r), \tag{3}$$

where $m_e$ denotes the electron mass, $\hbar$ stands for the Planck constant divided by $2\pi$ and the orbital angular momentum operator $\hat{\boldsymbol{l}}$ with its square $\hat{\boldsymbol{l}}^2$ is given by

$$\begin{aligned} \hat{\boldsymbol{l}} &= (\hat{l}_x, \hat{l}_y, \hat{l}_z) = (x,y,z) \times \frac{\hbar}{i}\nabla \\ &= \left(i\hbar\left[\sin\varphi\frac{\partial}{\partial\theta} + \cot\theta\cos\varphi\frac{\partial}{\partial\varphi}\right], i\hbar\left[-\cos\varphi\frac{\partial}{\partial\theta} + \cot\theta\sin\varphi\frac{\partial}{\partial\varphi}\right], -i\hbar\frac{\partial}{\partial\varphi}\right), \end{aligned} \tag{4}$$

$$\hat{\boldsymbol{l}}^2 = -\hbar^2\left[\frac{1}{\sin\theta}\frac{\partial}{\partial\theta}\left(\sin\theta\frac{\partial}{\partial\theta}\right) + \frac{1}{\sin^2\theta}\frac{\partial^2}{\partial\varphi^2}\right]. \tag{5}$$

Since $\hat{h}$, $\hat{\boldsymbol{l}}^2$ and $\hat{l}_z$ commute with each other, an eigenfunction $\psi_{nlm}(r,\theta,\varphi)$ of $\hat{h}$ can be written as

$$\psi_{nlm}(r,\theta,\varphi) = R_{nl}(r)Y_{lm}(\theta,\varphi), \tag{6}$$

where the complex spherical harmonics $Y_{lm}(\theta,\varphi)$ satisfy the following eigenvalue equations,

$$\begin{cases} \hat{\boldsymbol{l}}^2 Y_{lm}(\theta,\varphi) = l(l+1)\hbar^2 Y_{lm}(\theta,\varphi), & l = 0,1,2,\cdots, \\ \hat{l}_z Y_{lm}(\theta,\varphi) = m\hbar Y_{lm}(\theta,\varphi), & m = 0,\pm1,\pm2,\cdots,\pm l. \end{cases} \tag{7}$$

The substitution of $\psi_{nlm}(r,\theta,\varphi)$ into the Schrödinger equation,

$$\hat{h}\psi_{nlm}(r,\theta,\varphi) = \varepsilon_{nl}\psi_{nlm}(r,\theta,\varphi), \tag{8}$$

yields that the radial wave function $R_{nl}(r)$ obeys

$$-\frac{\hbar^2}{2m_e}\left[\frac{d^2R_{nl}(r)}{dr^2}+\frac{2}{r}\frac{dR_{nl}(r)}{dr}-\frac{l(l+1)}{r^2}R_{nl}(r)\right]+v(r)R_{nl}(r)=\varepsilon_{nl}R_{nl}(r), \tag{9}$$

where $\varepsilon_{nl}$ denotes the eigenenergy corresponding to $\psi_{nlm}(r,\theta,\varphi)$. From the last differential equation, $R_{nl}(r)$ is related to the $l$th order spherical Bessel function $j_l(kr)$ at $r\in[0,a]$,

$$R_{nl}(r)=j_l(kr),\quad k=\sqrt{\frac{2m_e\varepsilon_{nl}}{\hbar^2}}. \tag{10}$$

Because $R_{nl}(r)=0$ at $r\in(a,\infty)$, the boundary condition is $R_{nl}(a)=0$. Accordingly, $k$ is given by

$$k=\frac{X_{nl}}{a}, \tag{11}$$

where $X_{nl}$ is the $n$th smallest root of $j_l(\rho)=0$ with the unknown $\rho$. Finally, $\varepsilon_{nl}$ is obtained as

$$\varepsilon_{nl}=\frac{\hbar^2X_{nl}^2}{2m_ea^2}. \tag{12}$$

## Supplementary Note 2 Symmetry-Adapted Orbital Model

The structural symmetry of any metal cluster is lower than the spherical symmetry $O(3)$. We make symmetry-adapted linear combinations (SALCs) of the spherical jellium orbitals with respect to each symmetry. Combining such a symmetry adaptation with the lower-order perturbation theory, the level splittings of the spherical jellium orbitals in an actual metal cluster can be properly taken into consideration.

In the case of the structure belonging to a point group $G$, $\Lambda$-type electronic orbitals ($\Lambda : S(l=0), P(l=1), D(l=2), \cdots$) are subducted into irreducible representations $\Gamma_1, \Gamma_2, \cdots$ of $G$,

$$O(3) \downarrow G : \Lambda \rightarrow n_1\Gamma_1 \oplus n_2\Gamma_2 \oplus \cdots, \tag{13}$$

where $n_\mu$ stands for the number of $\Gamma_\mu$ which appears in this subduction. A subducted orbital $\psi_{n\Lambda(\Gamma\gamma)}$ with its irreducible representation $\Gamma$ and line $\gamma$, originating from the $n\Lambda$ spherical jellium orbitals, is given by

$$\psi_{n\Lambda(\Gamma\gamma)} = \sum_{m=-l}^{+l} C_{\Gamma\gamma lm}\psi_{nlm}(r,\theta,\varphi) = R_{nl}(r)\sum_{m=-l}^{+l} C_{\Gamma\gamma lm}Y_{lm}(\theta,\varphi), \tag{14}$$

where $C_{\Gamma\gamma lm}$ denotes a SALC coefficient. The mathematical forms of the subducted orbitals are explicitly shown later in the icosahedral, octahedral and tetrahedral cases.

Let us subsequently consider the energy level $\varepsilon_{n\Lambda(\Gamma)}$ of a subducted orbital $\psi_{n\Lambda(\Gamma\gamma)}$ under a perturbation $\Delta v$ due to nuclear charge distribution of each cluster. According to the first-order perturbation theory, $\varepsilon_{n\Lambda(\Gamma)}$ is written by

$$\varepsilon_{n\Lambda(\Gamma)} = \varepsilon^{(0)}_{n\Lambda(\Gamma)} + \varepsilon^{(1)}_{n\Lambda(\Gamma)}, \tag{15}$$

where the zeroth-order energy $\varepsilon^{(0)}_{n\Lambda(\Gamma)}$ is a spherical jellium orbital energy,

$$\varepsilon^{(0)}_{n\Lambda(\Gamma)} = \frac{\hbar^2 X_{nl}^2}{2m_e a^2}, \tag{16}$$

and the first-order energy correction $\varepsilon^{(1)}_{n\Lambda(\Gamma)}$ is given by

$$\varepsilon^{(1)}_{n\Lambda(\Gamma)} = \iiint \left( \left| \psi_{n\Lambda(\Gamma\gamma)} \right|^2 \Delta v \right) r^2 \sin\theta dr d\theta d\varphi. \tag{17}$$

The first-order correction selectively stabilizes a subducted orbital overlapping the nuclear charge distribution in a cluster (see the integrand). If the higher-order perturbation theory is applied, the mixing effect between the subducted orbitals with the same irreducible representation can also be taken into account.

## Supplementary Note 2.1 Icosahedral Case

$$\psi_{n\Lambda(\Gamma\gamma)} = R_{nl}(r) \times \sum_{m=-l}^{+l} C_{\Gamma\gamma lm} Y_{lm}(\theta,\varphi)$$

$$\psi_{nS(a_{1g}\gamma)} = R_{n,0}(r) \times Y_{0,0}(\theta,\varphi) \qquad (\gamma=1) \tag{18}$$

$$\psi_{nP(t_{1u}\gamma)} = R_{n,1}(r) \times \begin{cases} Y_{1,+1}(\theta,\varphi) & (\gamma=1) \\ Y_{1,0}(\theta,\varphi) & (\gamma=2) \\ Y_{1,-1}(\theta,\varphi) & (\gamma=3) \end{cases} \tag{19}$$

$$\psi_{nD(h_g\gamma)} = R_{n,2}(r) \times \begin{cases} Y_{2,+2}(\theta,\varphi) & (\gamma=1) \\ Y_{2,+1}(\theta,\varphi) & (\gamma=2) \\ Y_{2,0}(\theta,\varphi) & (\gamma=3) \\ Y_{2,-1}(\theta,\varphi) & (\gamma=4) \\ Y_{2,-2}(\theta,\varphi) & (\gamma=5) \end{cases} \tag{20}$$

$$\psi_{nF(t_{2u}\gamma)} = R_{n,3}(r) \times \begin{cases} \sqrt{\frac{3}{32}}\left(\sqrt{5}-1\right) Y_{3,+1}(\theta,\varphi) - \sqrt{\frac{1}{32}}\left(\sqrt{5}+3\right) Y_{3,+3}(\theta,\varphi) & (\gamma=1) \\ \sqrt{\frac{1}{4}} Y_{3,0}(\theta,\varphi) + \sqrt{\frac{3}{4}} Y_{3,+2}(\theta,\varphi) & (\gamma=2) \\ \sqrt{\frac{3}{32}}\left(\sqrt{5}+1\right) Y_{3,-1}(\theta,\varphi) - \sqrt{\frac{1}{32}}\left(\sqrt{5}-3\right) Y_{3,-3}(\theta,\varphi) & (\gamma=3) \end{cases} \tag{21}$$

$$\psi_{nF(g_u\gamma)} = R_{n,3}(r) \times \begin{cases} Y_{3,-2}(\theta,\varphi) & (\gamma=1) \\ \sqrt{\frac{1}{32}}\left(\sqrt{5}+3\right) Y_{3,+1}(\theta,\varphi) + \sqrt{\frac{3}{32}}\left(\sqrt{5}-1\right) Y_{3,+3}(\theta,\varphi) & (\gamma=2) \\ \sqrt{\frac{3}{4}} Y_{3,0}(\theta,\varphi) - \sqrt{\frac{1}{4}} Y_{3,+2}(\theta,\varphi) & (\gamma=3) \\ \sqrt{\frac{1}{32}}\left(\sqrt{5}-3\right) Y_{3,-1}(\theta,\varphi) + \sqrt{\frac{3}{32}}\left(\sqrt{5}+1\right) Y_{3,-3}(\theta,\varphi) & (\gamma=4) \end{cases} \tag{22}$$

## Supplementary Note 2.2 Octahedral Case

$$\psi_{n\Lambda(\Gamma\gamma)} = R_{nl}(r) \times \sum_{m=-l}^{+l} C_{\Gamma\gamma lm} Y_{lm}(\theta,\varphi)$$

$$\psi_{nS(a_{1g}\gamma)} = R_{n,0}(r) \times Y_{0,0}(\theta,\varphi) \qquad (\gamma=1) \tag{23}$$

$$\psi_{nP(t_{1u}\gamma)} = R_{n,1}(r) \times \begin{cases} Y_{1,+1}(\theta,\varphi) & (\gamma=1) \\ Y_{1,0}(\theta,\varphi) & (\gamma=2) \\ Y_{1,-1}(\theta,\varphi) & (\gamma=3) \end{cases} \tag{24}$$

$$\psi_{nD(e_g\gamma)} = R_{n,2}(r) \times \begin{cases} Y_{2,+2}(\theta,\varphi) & (\gamma=1) \\ Y_{2,0}(\theta,\varphi) & (\gamma=2) \end{cases} \tag{25}$$

$$\psi_{nD(t_{2g}\gamma)} = R_{n,2}(r) \times \begin{cases} Y_{2,+1}(\theta,\varphi) & (\gamma=1) \\ Y_{2,-1}(\theta,\varphi) & (\gamma=2) \\ Y_{2,-2}(\theta,\varphi) & (\gamma=3) \end{cases} \tag{26}$$

$$\psi_{nF(a_{2u}\gamma)} = R_{n,3}(r) \times Y_{3,-2}(\theta,\varphi) \qquad (\gamma=1) \tag{27}$$

$$\psi_{nF(t_{1u}\gamma)} = R_{n,3}(r) \times \begin{cases} \sqrt{\frac{3}{8}} Y_{3,+1}(\theta,\varphi) + \sqrt{\frac{5}{8}} Y_{3,+3}(\theta,\varphi) & (\gamma=1) \\ Y_{3,0}(\theta,\varphi) & (\gamma=2) \\ \sqrt{\frac{3}{8}} Y_{3,-1}(\theta,\varphi) - \sqrt{\frac{5}{8}} Y_{3,-3}(\theta,\varphi) & (\gamma=3) \end{cases} \tag{28}$$

$$\psi_{nF(t_{2u}\gamma)} = R_{n,3}(r) \times \begin{cases} \sqrt{\frac{5}{8}} Y_{3,+1}(\theta,\varphi) - \sqrt{\frac{3}{8}} Y_{3,+3}(\theta,\varphi) & (\gamma=1) \\ Y_{3,+2}(\theta,\varphi) & (\gamma=2) \\ \sqrt{\frac{5}{8}} Y_{3,-1}(\theta,\varphi) + \sqrt{\frac{3}{8}} Y_{3,-3}(\theta,\varphi) & (\gamma=3) \end{cases} \tag{29}$$

## Supplementary Note 2.3 Tetrahedral Case

$$\psi_{n\Lambda(\Gamma\gamma)} = R_{nl}(r) \times \sum_{m=-l}^{+l} C_{\Gamma\gamma lm} Y_{lm}(\theta,\varphi)$$

$$\psi_{nS(a_1\gamma)} = R_{n,0}(r) \times Y_{0,0}(\theta,\varphi) \qquad (\gamma=1) \tag{30}$$

$$\psi_{nP(t_2\gamma)} = R_{n,1}(r) \times \begin{cases} Y_{1,+1}(\theta,\varphi) & (\gamma=1) \\ Y_{1,0}(\theta,\varphi) & (\gamma=2) \\ Y_{1,-1}(\theta,\varphi) & (\gamma=3) \end{cases} \tag{31}$$

$$\psi_{nD(e\gamma)} = R_{n,2}(r) \times \begin{cases} Y_{2,+2}(\theta,\varphi) & (\gamma=1) \\ Y_{2,0}(\theta,\varphi) & (\gamma=2) \end{cases} \tag{32}$$

$$\psi_{nD(t_2\gamma)} = R_{n,2}(r) \times \begin{cases} Y_{2,+1}(\theta,\varphi) & (\gamma=1) \\ Y_{2,-1}(\theta,\varphi) & (\gamma=2) \\ Y_{2,-2}(\theta,\varphi) & (\gamma=3) \end{cases} \tag{33}$$

$$\psi_{nF(a_1\gamma)} = R_{n,3}(r) \times Y_{3,-2}(\theta,\varphi) \qquad (\gamma=1) \tag{34}$$

$$\psi_{nF(t_1\gamma)} = R_{n,3}(r) \times \begin{cases} \sqrt{\frac{5}{8}} Y_{3,+1}(\theta,\varphi) - \sqrt{\frac{3}{8}} Y_{3,+3}(\theta,\varphi) & (\gamma=1) \\ Y_{3,+2}(\theta,\varphi) & (\gamma=2) \\ \sqrt{\frac{5}{8}} Y_{3,-1}(\theta,\varphi) + \sqrt{\frac{3}{8}} Y_{3,-3}(\theta,\varphi) & (\gamma=3) \end{cases} \tag{35}$$

$$\psi_{nF(t_2\gamma)} = R_{n,3}(r) \times \begin{cases} \sqrt{\frac{3}{8}} Y_{3,+1}(\theta,\varphi) + \sqrt{\frac{5}{8}} Y_{3,+3}(\theta,\varphi) & (\gamma=1) \\ Y_{3,0}(\theta,\varphi) & (\gamma=2) \\ \sqrt{\frac{3}{8}} Y_{3,-1}(\theta,\varphi) - \sqrt{\frac{5}{8}} Y_{3,-3}(\theta,\varphi) & (\gamma=3) \end{cases} \tag{36}$$

## Supplementary Figures

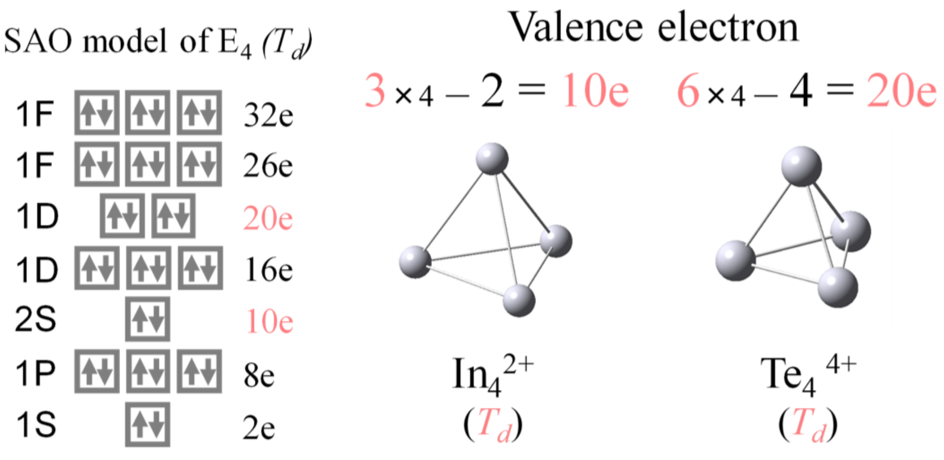


Supplementary Figure 1: The optimized geometries of $[In_4]^{2+}$ and $[Te_4]^{4+}$ clusters. The SAO-based electronic configuration is also shown for reference.

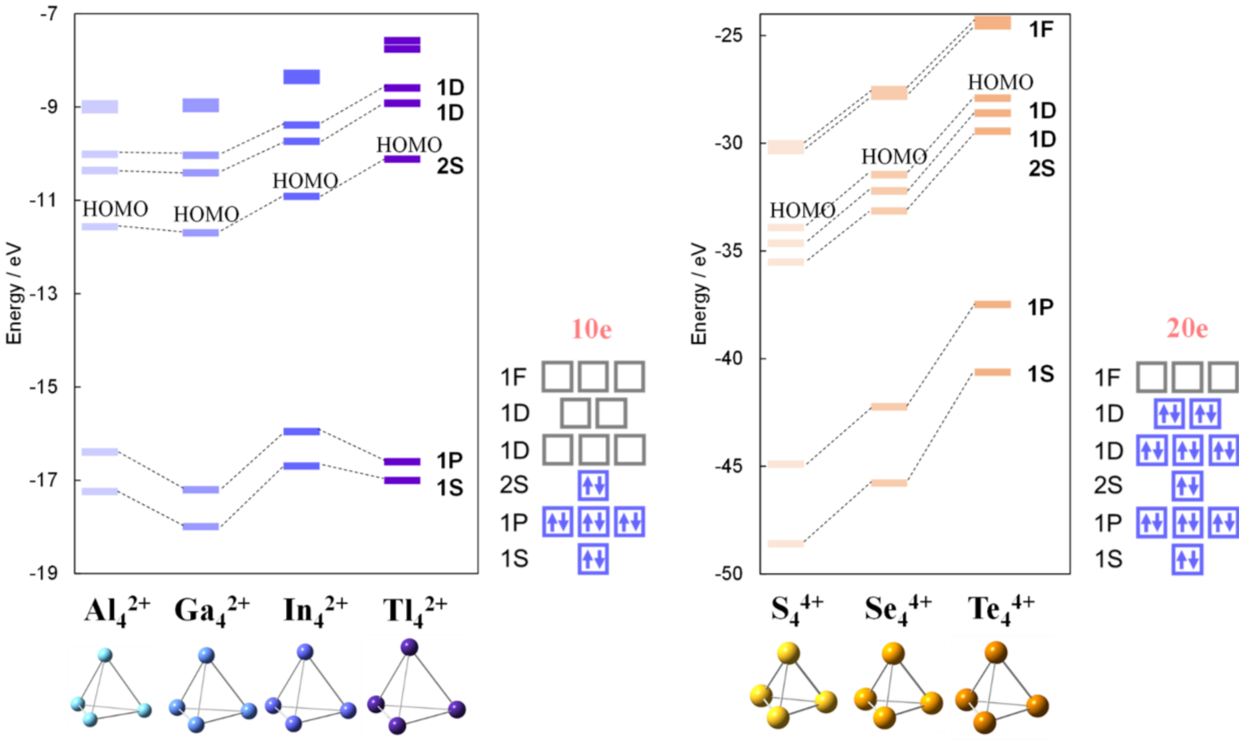


Supplementary Figure 2: The molecular orbital levels of $[Al_4]^{2+}$, $[Ga_4]^{2+}$, $[In_4]^{2+}$ and $[Tl_4]^{2+}$ (left) and those of $[S_4]^{4+}$, $[Se_4]^{4+}$ and $[Te_4]^{4+}$ (right). The SAO-based electronic configurations are also shown for reference.

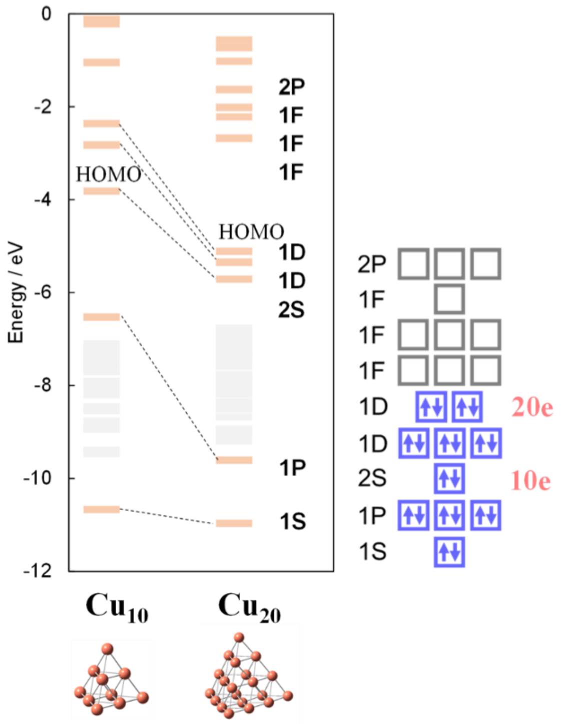


Supplementary Figure 3: The optimized geometries and molecular orbital levels of $Cu_{10}$ and $Cu_{20}$. The SAO-based electronic configuration is also shown for reference.

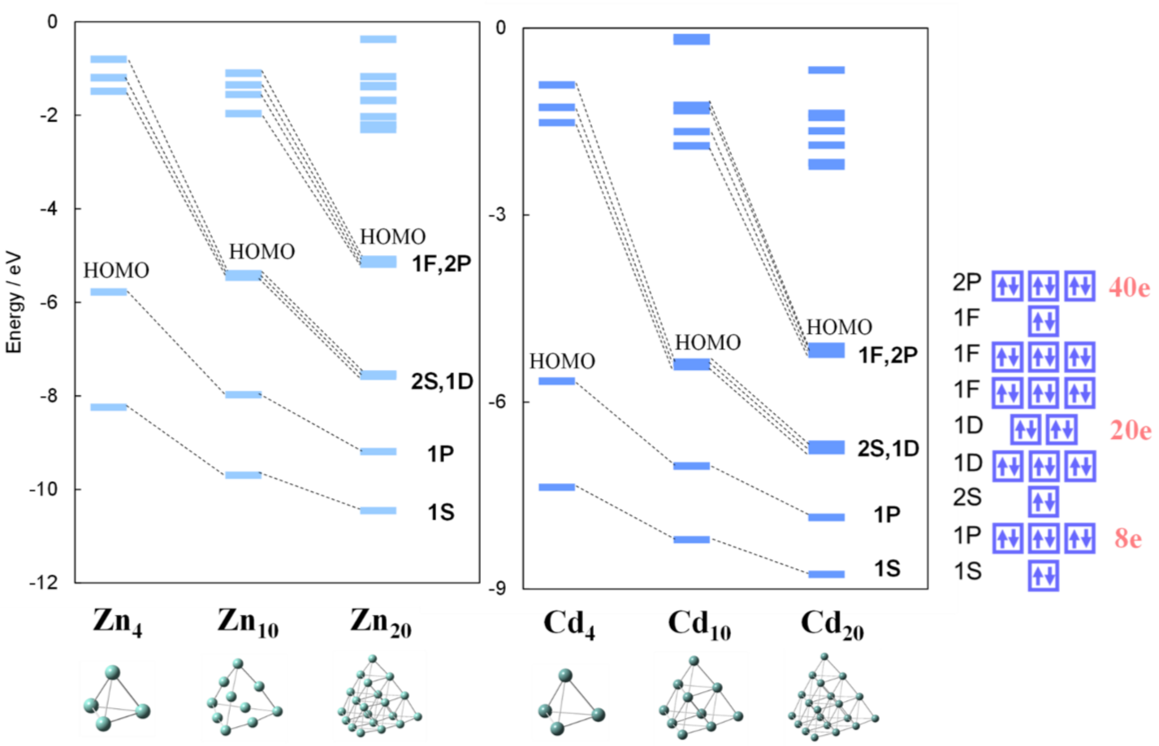


Supplementary Figure 4: The optimized geometries and molecular orbital levels of $Zn_4$, $Zn_{10}$ and $Zn_{20}$ (left) and those of $Cd_4$, $Cd_{10}$ and $Cd_{20}$ (right). The SAO-based electronic configuration is also shown for reference.

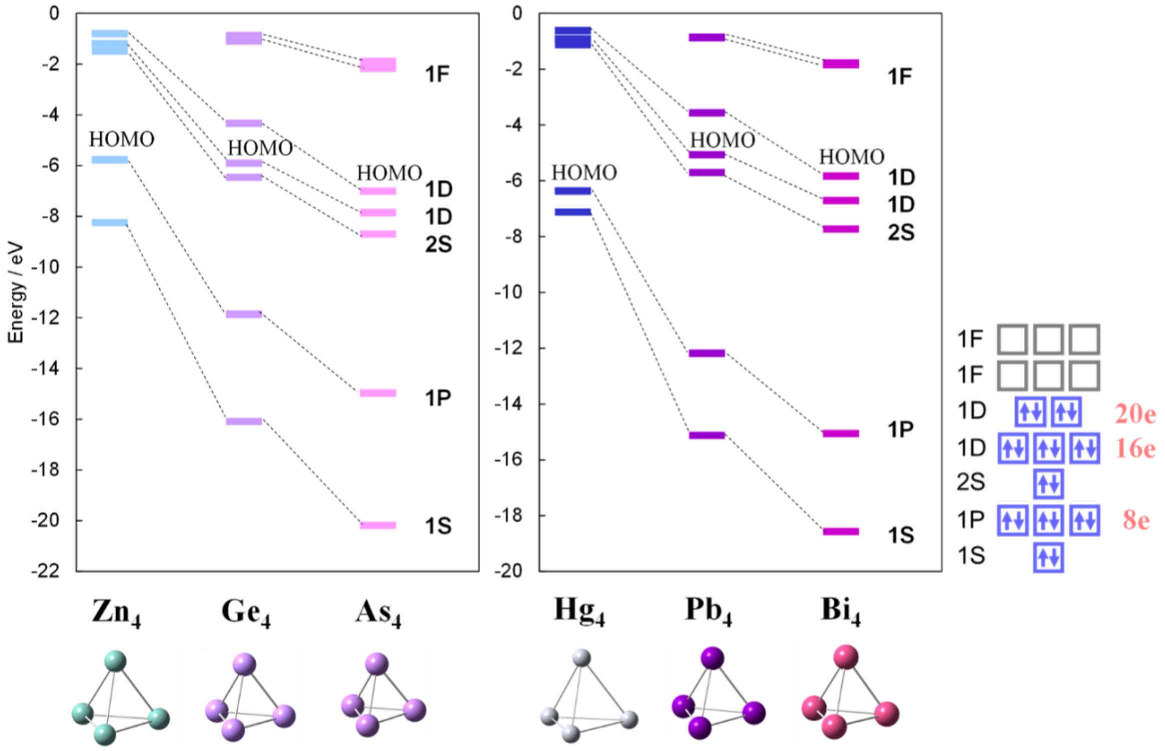


Supplementary Figure 5: The optimized geometries and molecular orbital levels of $Zn_4$, $Ge_4$ and $As_4$ (left) and those of $Hg_4$, $Pb_4$ and $Bi_4$ (right). The SAO-based electronic configuration is also shown for reference.

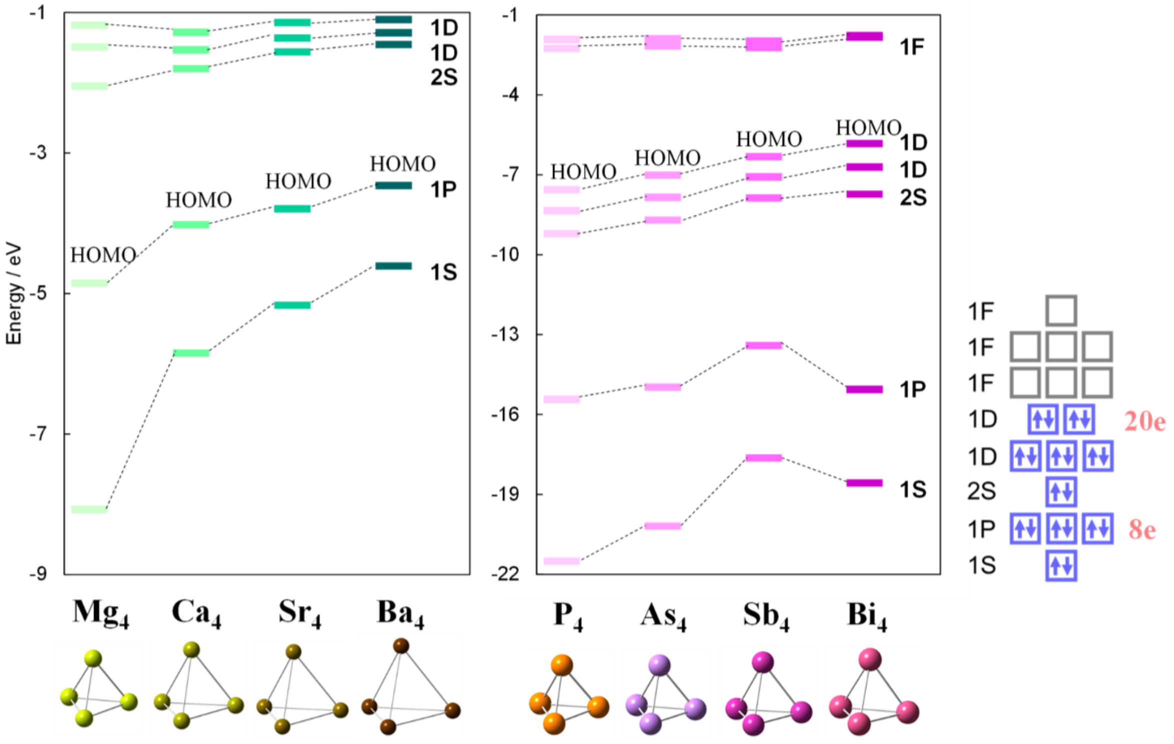


Supplementary Figure 6: The optimized geometries and molecular orbital levels of $Mg_4$, $Ca_4$, $Sr_4$ and $Ba_4$ (left) and those of $P_4$, $As_4$, $Sb_4$ and $Bi_4$ (right). The SAO-based electronic configuration is also shown for reference.

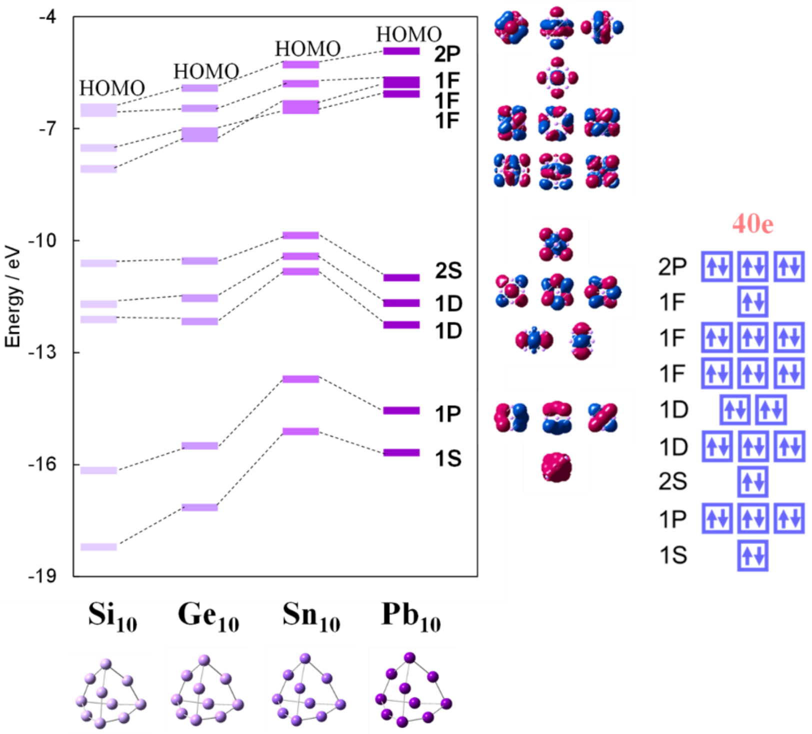


Supplementary Figure 7: The optimized geometries and molecular orbital levels of $Si_{10}$, $Ge_{10}$, $Sn_{10}$ and $Pb_{10}$. The SAO-based electronic configuration is also shown for reference.

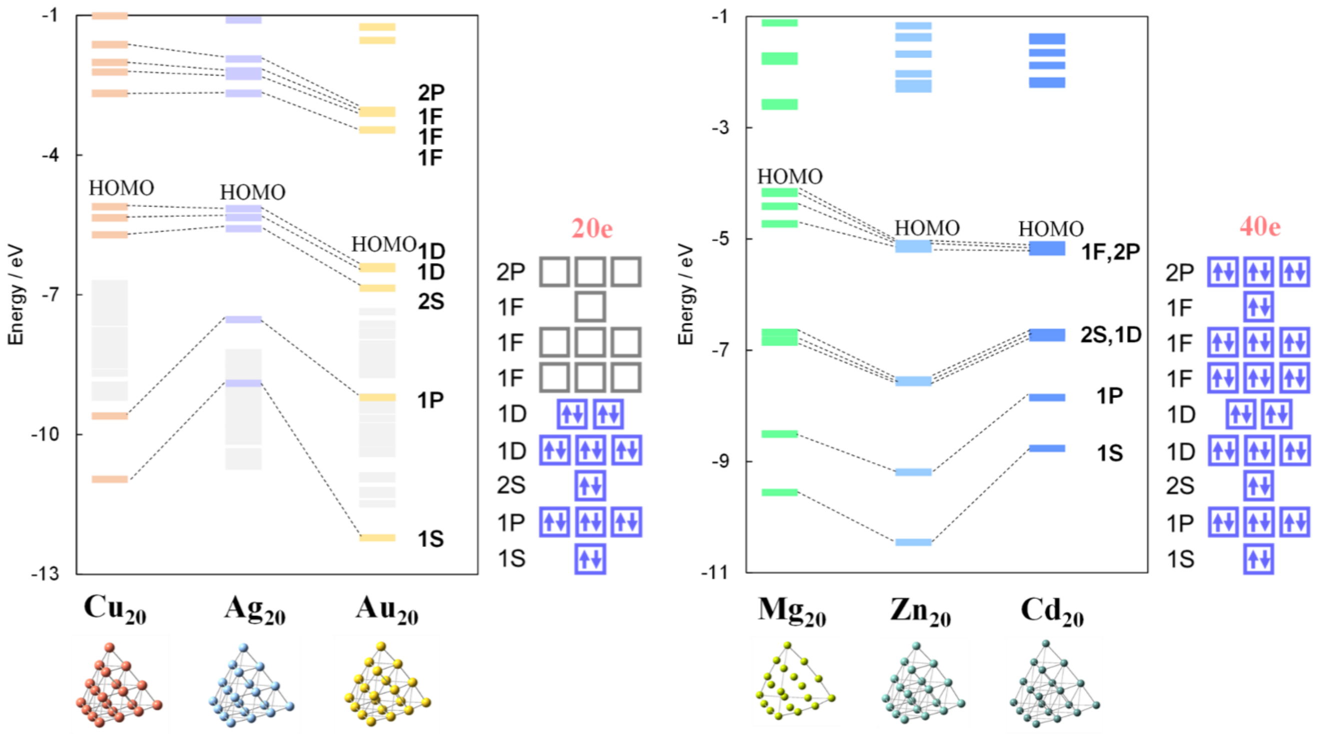


Supplementary Figure 8: The optimized geometries and molecular orbital levels of $Cu_{20}$, $Ag_{20}$ and $Au_{20}$ (left) and those of $Mg_{20}$, $Zn_{20}$ and $Cd_{20}$ (right). The SAO-based electronic configurations are also shown for reference.

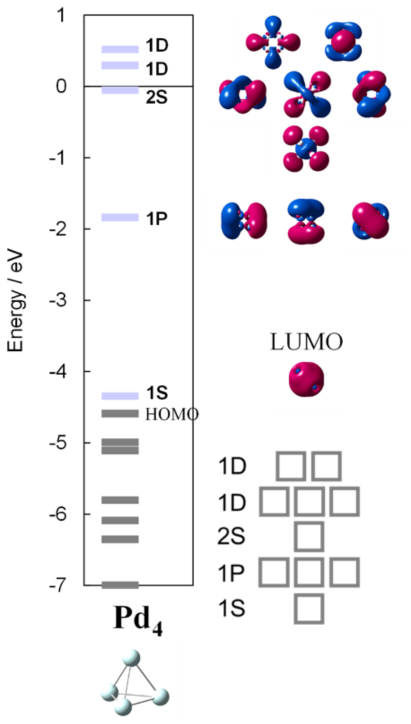


Supplementary Figure 9: The optimized geometry and molecular orbital levels of $Pd_4$. The SAO-based electronic configuration is also shown for reference.

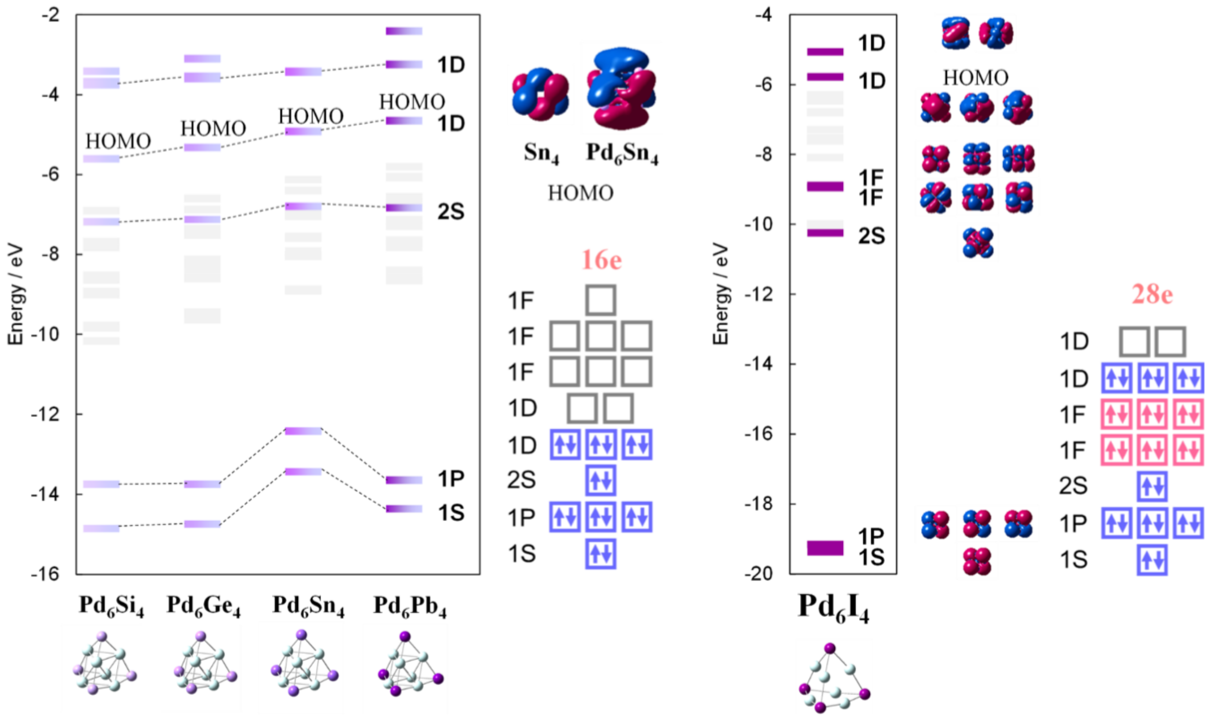


Supplementary Figure 10: The optimized geometries and molecular orbital levels of $Pd_6Si_4$, $Pd_6Ge_4$, $Pd_6Sn_4$ and $Pd_6Pb_4$ (left) and those of $Pd_6I_4$ (right). The SAO-based electronic configurations are also shown for reference.

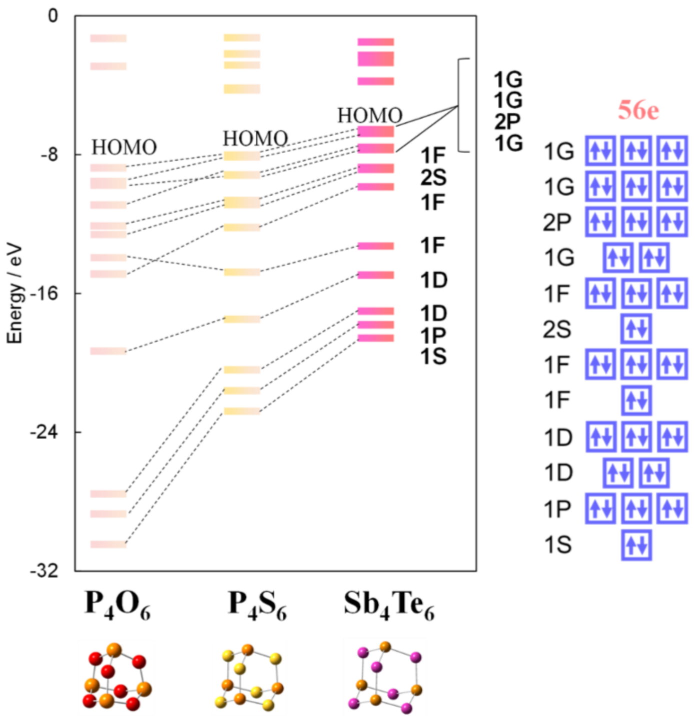


Supplementary Figure 11: The optimized geometries and molecular orbital levels of $P_4O_6$, $P_4S_6$ and $Sb_4Te_6$. It should also be noted that many valence electron systems, such as oxygen- and sulfur-bridged clusters, have strong interelectronic interactions and distorted structures, often changing the order of orbital levels partially, as compared with Fig. 1.

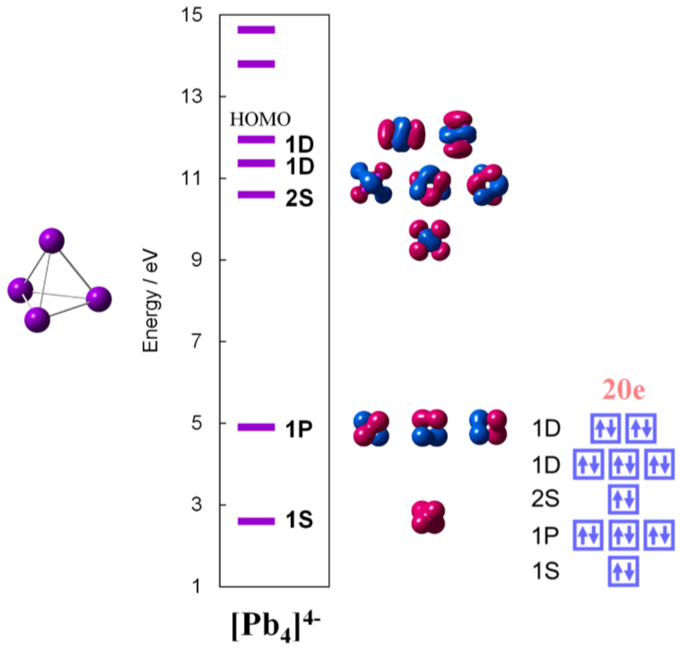


Supplementary Figure 12: The optimized geometry and molecular orbital levels of $[Pb_4]^{4-}$. The SAO-based electronic configuration is also shown for reference.

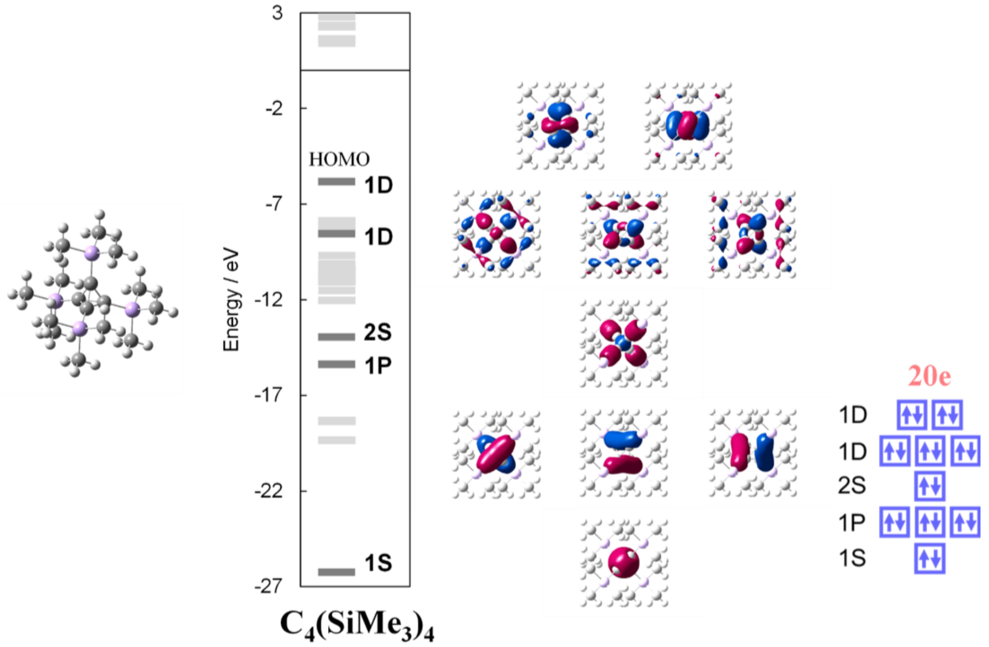


Supplementary Figure 13: The optimized geometry and molecular orbital levels of $C_4(SiMe_3)_4$. The SAO-based electronic configuration is also shown for reference.

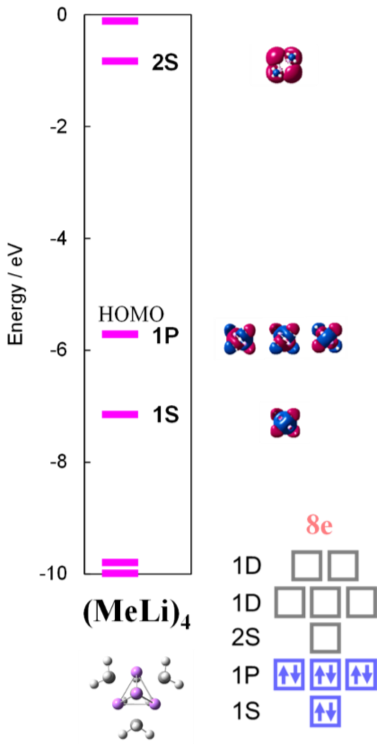


Supplementary Figure 14: The optimized geometry and molecular orbital levels of $(MeLi)_4$. The SAO-based electronic configurations are also shown for reference.

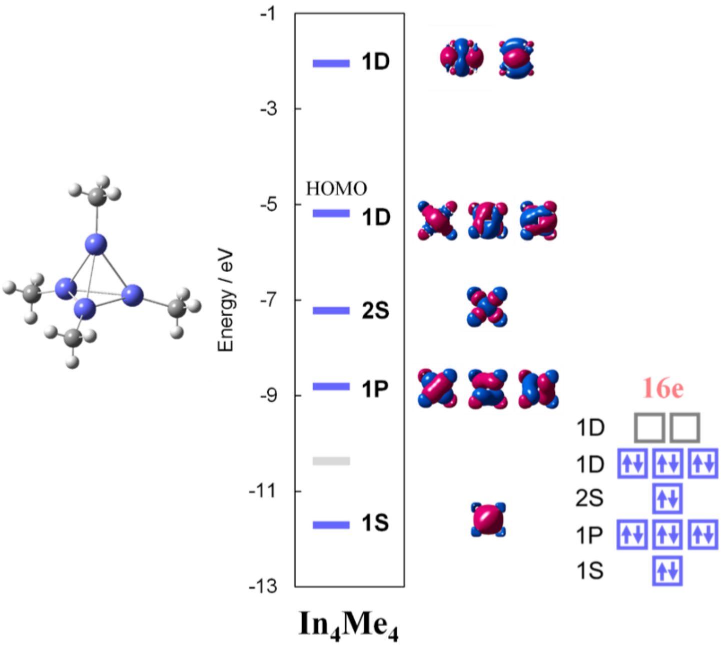


Supplementary Figure 15: The optimized geometry and molecular orbital levels of $In_4Me_4$. The SAO-based electronic configuration is also shown for reference.

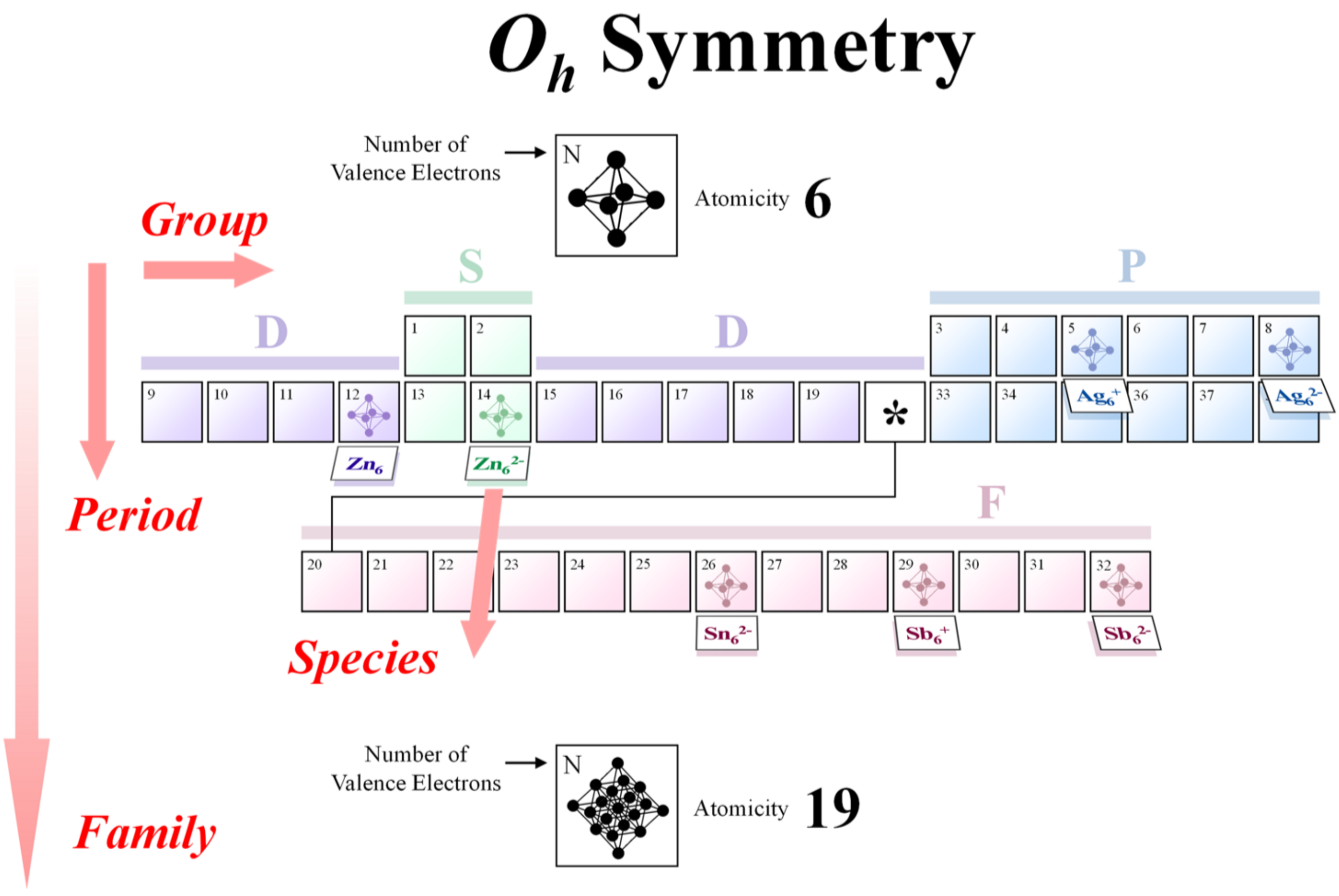


Supplementary Figure 16: The periodic framework based on the $O_h$-type SAO model.

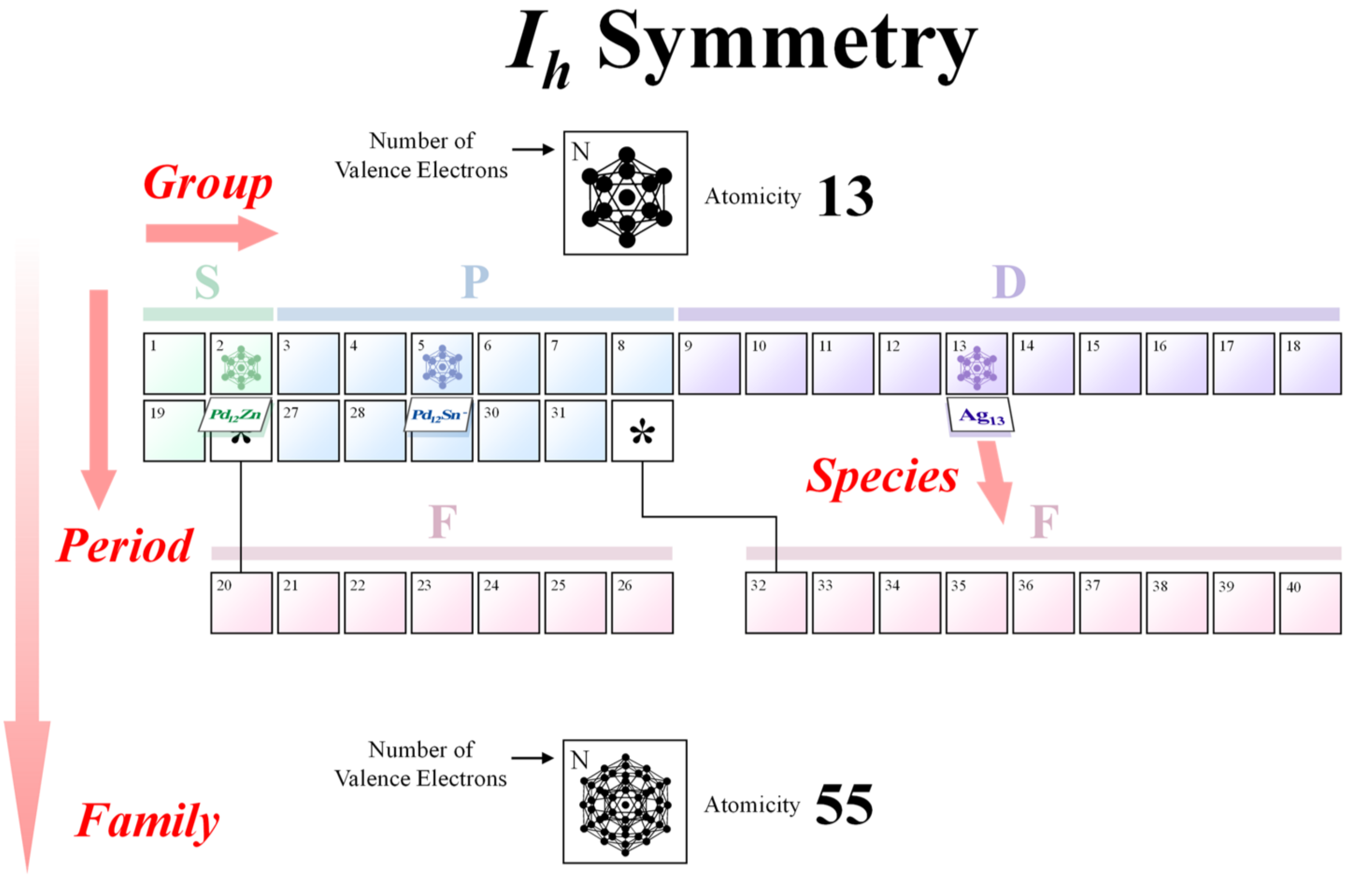


Supplementary Figure 17: The periodic framework based on the $I_h$-type SAO model.

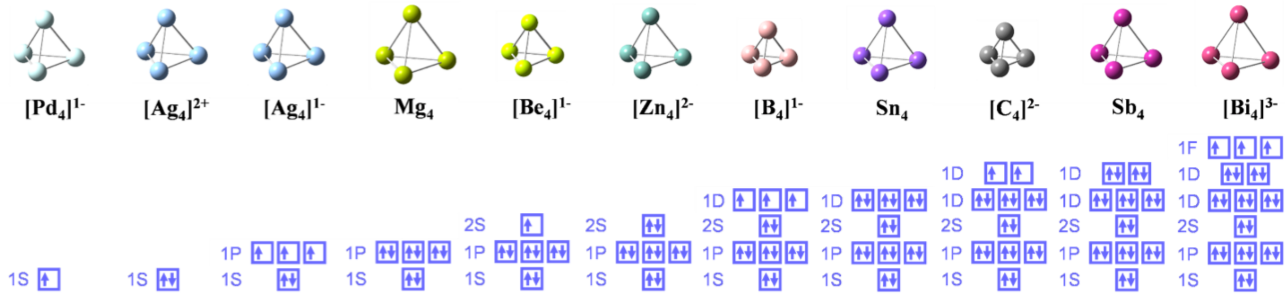


Supplementary Figure 18: The optimized geometries and SAO-based electronic configurations of 4-atom tetrahedral clusters.

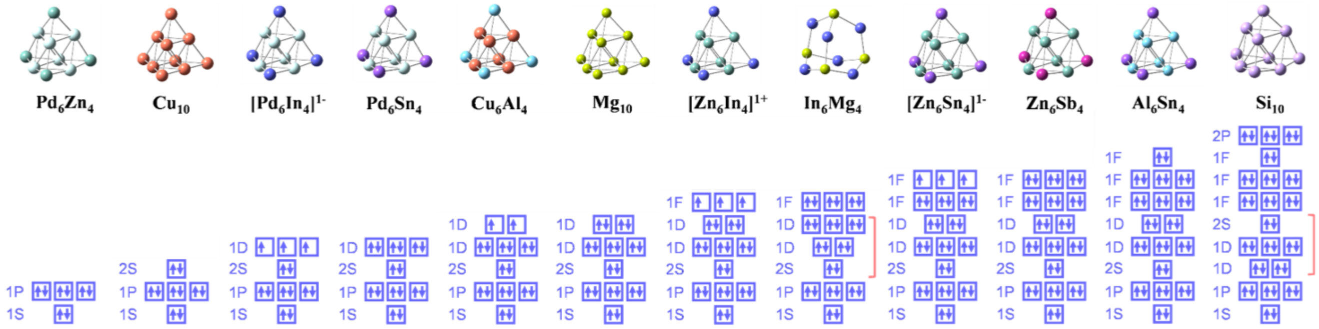


Supplementary Figure 19: The optimized geometries and SAO-based electronic configurations of 10-atom tetrahedral clusters.

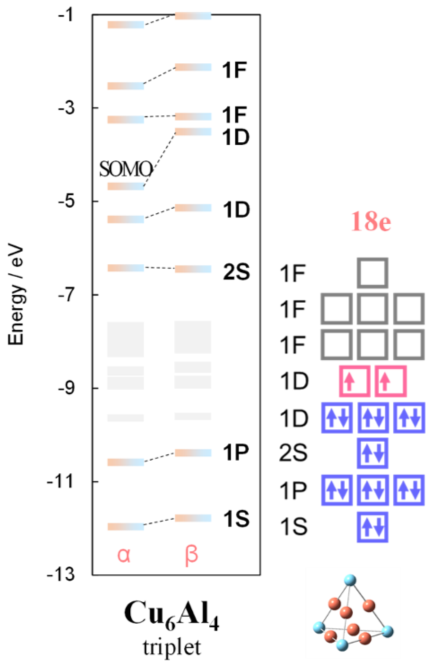


Supplementary Figure 20: The optimized geometry and molecular orbital levels of $Cu_6Al_4$ in the triplet state. The SAO-based electronic configuration is also shown for reference.

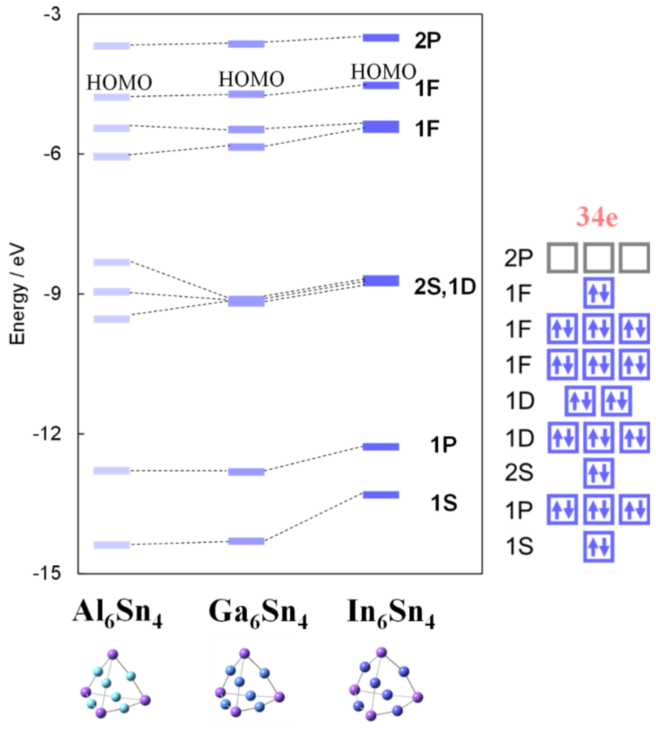


Supplementary Figure 21: The optimized geometries and molecular orbital levels of $Al_6Sn_4$, $Ga_6Sn_4$ and $In_6Sn_4$. The SAO-based electronic configuration is also shown for reference.
